# International chemical identifier for reactions (RInChI)

**DOI:** 10.1186/1758-2946-5-45

**Published:** 2013-10-24

**Authors:** Guenter Grethe, Jonathan M Goodman, Chad HG Allen

**Affiliations:** 1 352 Channing Way, Alameda, CA 94502, USA; 2 Department of Chemistry, University of Cambridge, Cambridge CB2 1EW, UK

## Abstract

The IUPAC International Chemical Identifier (InChI) provides a method to generate a unique text descriptor of molecular structures. Building on this work, we report a process to generate a unique text descriptor for reactions, RInChI. By carefully selecting the information that is included and by ordering the data carefully, different scientists studying the same reaction should produce the same RInChI. If differences arise, these are most likely the minor layers of the InChI, and so may be readily handled. RInChI provides a concise description of the key data in a chemical reaction, and will help enable the rapid searching and analysis of reaction databases.

## Background

Since its inception, the IUPAC International Chemical Identifier (InChI) [1,2] has found wide acceptance as a standard in the chemical community. In order to widen the applicability of the identifier, the IUPAC Division VIII Subcommittee and the InChI Trust [3] have initiated several projects to extend the usage of the identifier. Among these is the development of a non-proprietary, international identifier for reactions (RInChI) [4] to describe chemical reactions in a unique machine-readable character string based on the InChI algorithm suitable for data storage and indexing. For this purpose, a working group was established in 2008 and the initial developmental work was carried out at Cambridge University under the supervision of Jonathan Goodman resulting in a preliminary working version of the program. This note is an interim report based on the discussions of the working group, the work on the project carried out by Chad Allen [5] and others in the Goodman group and a presentation by Guenter Grethe at the 8^th^ German Conference on Chemoinformatics [6]. Further work will be carried out before publication of the RInChI standard.

## Introduction

A number of methods are available to represent molecular structures as a single line of text. The most commonly used of these are SMILES, developed by Daylight Chemical Information Systems, Inc, [7] and the IUPAC International Chemical Identifier (InChI). Different researchers investigating the same molecular structure, should be able to write down the same InChI and the same canonical SMILES without needing to consult each other. It would be very useful to be able to do the same thing for reactions. However, comparing reactions is much more challenging than comparing structures as more information is available and decisions have to be made which aspects of this information must be stored.

Daylight [7] has developed SMILES so that they can be used to describe reactions and SMILES to describe transformations [7]. The Sybyl Line Notation (SLN) [8] can also be used to represent chemical reactions in a line notation. Both of these approaches are powerful and flexible, permitting the inclusion of a range of information including atom-mapping. Both are excellent tools to describe reactions. However, different researchers studying the same reaction may well select different data to include in the line notation, and so generate different descriptions of one reaction.

The objective of the RInChI project is the creation of an unambiguous description for reactions from their structural diagrams, Rxn- and RDfiles for which different researchers should, so far as possible, generate the same identifier for the same reaction. The generated identifier will allow the organization and validation of new reaction databases and will enable the comparison of different data sources. In line with the multi-layer concept of InChI, the basic RInChI in addition to the InChIs of reactants, products, solvents, and catalysts must include information about equilibrium, unbalanced or multi-step reactions. Furthermore, the format of the identifier has to be open to include future information, such as reaction conditions and non-unique molecular entities. Since the identifier can be quite long depending on the number of participating molecules, long and short versions of RInChIKeys were developed. The RInChI project software is implemented as an importable Python package, including usage scripts for conversion, addition and analysis.

## RInChI format

### Full RInChI string

Analogous to InChI, the RInChI format is a hierarchical, layered description of a reaction with different levels. The RInChI of version 0.02 includes the RInChI label, three groups of molecules and further information layers.

**Figure 1:**
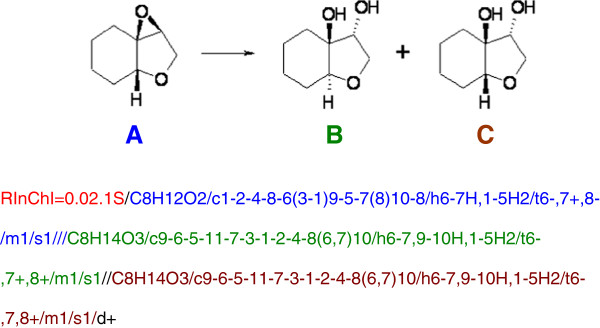
**RInChI format: Individual InChIs are identified in color, the directional label is black.** The colors are not part of the RInChI, and are included here only to highlight the different parts of the string.

The label starts with the acronym RInChI, followed by the RInChI version number and the InChI version number used to generate molecule InChIs separated by a period. In the example shown in Figure 1, the label reads RInChI = 0.02.1S, *i.e.* the RInChI version is 0.02 and the InChI version is 1S. The RInChI version number will always have exactly one decimal point.

Three groups of molecules are described in the RInChI identifier, one group for each side of the arrow and one group of molecules which are above, below or on both sides of the arrow, *i.e.* solvents and catalysts. Each group is described as a list of InChIs which are sorted within a group. After sorting the molecules within a group, the groups representing starting materials and products are sorted using the unix 'sort’ command. Valid RInChIs do not require all three groups to be present. For example, a RInChI of a reaction without a known product and no information about solvents/reagents would only show the first group. Individual InChIs within a group are separated by a double slash “//” and the groups of molecules are separated by a triple slash “///”. Since the display of the first two groups in a RInChI does not indicate which one represents reactants or products, directionality is shown by an additional layer: “/d+” indicates that reactants are followed by products, “/d-“ represents the reverse direction and “/d=” represents an equilibrium reaction. Additional layers, for example information about reaction conditions, might be added in future versions of the program.

For example, the reaction: **1** → **2** catalysed by **3**, would be represented by the RInChI:

RInChI=0.02.1.S/group1///group2///group3/

Here *group1*, *group2*, *group3* are the list of InChIs in **1**, **2** and **3** respectively. If the starting material, **1**, includes several molecules, they would be listed in the order defined by the unix 'sort’ command, and separated by a double slash: “//”. Similarly, *group2* may include several different products, and *group3* may include several catalysts and other substances which are present both at the beginning and end of the reaction, such as solvents.

The order of *group1* and *group2* is determined by the unix 'sort’ command. The RInChI as written above, does not distinguish between **1** → **2** and **2** → **1**. This is because the direction of many reactions, such as acetal formation/hydrolysis, is decided by the details of the conditions rather than the reagents. The direction of the reaction can be indicated by a layer at the end of the RInChI: “/d + ”, “/d-” or “/d = ”.

In this example (Figure 1), *group1* is molecule **A**, *group2* is molecules **B** and **C**, and *group3* is omitted as the reaction diagram does not include any information about solvents or catalysts. The direction of the reaction is indicated by the “/d + ” at the end of the string. The starting material is in *group1* and the products are in *group2*, because the starting material InChIs are sorted before the product InChIs by the unix 'sort’ command. Roughly 50% of RInChIs for which directionality is defined are expected to have the products in *group1* and the starting materials in *group2*. This is indicated in the RInChI by the use of “/d-” in the final layer. However, there are likely to be many RInChIs which represent equilibria with no preferred direction, or else reactions for which the directionality is uncertain. In the latter case, a RInChI should be used in which the direction layer is omitted, and such a string is a valid RInChI.

### RInChIKeys

Since full RInChI strings can be very long, it is useful to have access to a shortened version. RInChIKeys are hashed representations of the parent RInChIs. They are not backwards-convertible. However, they are useful for database manipulations. Two different types of RInChIKeys were developed, a composite of individual InChIKeys (long form) and a hashed digest of the RInChI as a whole (short form). Each type is available in two versions (A and B), with the latter containing additional information. We expect version B to be more useful in both cases.

The RInChIKeys comprise sequences of letters separated by hyphens. We refer to each sequence as a 'block’.

#### Long RInChIKey

In the long RInChIKey all molecules in the reaction are encoded as separate InChIKeys and grouped similar to the grouping of InChIs in RInChIs. This process results in variable length of the key depending on the number of molecules in the reaction.

##### Version A

**Figure 2:**
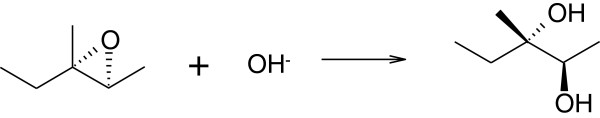
**Long RInChIKey, versions A and B. A**. Long RInChIKey = aSA-**EFKSL**-ZISUZIXPPXXNPC-WDSKDSIN-N-XLYOFNOQVPJJNP-UHFFFAOY-M--RLWWHEFTJSHFRN-RITPCOAN-N. **B**. Long RInChIKey = aSA-**FEANN**-ZISUZIXPPXXNPC-WDSKDSIN-N-XLYOFNOQVPJJNP-UHFFFAOY-M--RLWWHEFTJSHFRN-RITPCOAN-N.

As shown in Figure 2A, the first block (group of letters) consists of three letters of which the first one represents the version identifier and the next two identify the constituent InChIKeys. The second block, which is separated from the first block with a hypen, is a hashed representation of any additional reaction layers taken as a whole. The following blocks are groups of InChIKeys for all of the molecules in the RInChI following the same order as the molecules in the original RInChI. The division between the groups, which is indicated by a triple slash “///” in the RInChI, is marked in the RInChIKey by a double hyphen.

The directional information in the RInChI, if present, is encoded in block 2 and cannot be extracted from the RInChIKey.

##### Version B

Because the directional information may be useful, we also developed Version B of the long RInChIKey. In this version, the first letter of block 2 is F, B, E or U representing forward, backward, equilibrium, or unspecified reactions, respectively. The reminder of block 2 is a hash of the remaining additional reaction layer information. The directional information now allows identifying or searching for sets of reactants, products or agents. All the other blocks are identical in versions A and B.

#### Short RInChIKeys

**Figure 3:**
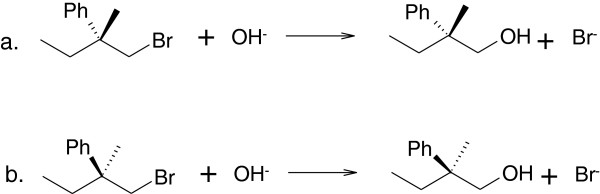
**Short RInChIKeys, version A, for a pair of enantiomeric reactions. a**. Short RInChIKey = aSA-BQLJB-IHEAXHQDHHDVWSD-BDQFIIFTLXHFxWF-EANNATPGMBMFBIQ. **b**. Short RInChIKey = aSA-BQLJB-DFULJJCJAAOPALL-BIJIEHNYVARRSAG-EANNATPGMBMFBIQ.

The length of a long RInChIKey varies with the number of molecules included in the RInChI. For some purposes, a fixed length key is preferable, even though it can encode less information. We have, therefore, also developed short RInChIKeys which are fixed-length, hashed representations of RInChIs. They are generated directly from the RInChIs and do not use the InChIKeys of individual molecular structures. Examples for both versions of the RInChIKey are shown in Figure 3.

##### Version A

This version encodes the groups of structures in a RInChI as simple entities and use the naïve hash described for version A of the long RInChIKey for the reaction layers, thereby neglecting the layered character of the InChIs. The first two blocks are the same as the first two blocks of the long RInChIKey. These are followed by exactly three more blocks, which encode the three groups of molecules in the original RInChI. These blocks are present even if the group is empty. This leads to completely different reactant and product blocks for the two enantiomers shown in Figure 3. Note that the fifth block, corresponding to *group3* is the same for both, because it is empty for both reactions.

##### Version B

**Figure 4:**
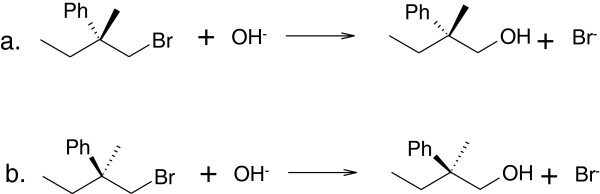
**Short RInChIKeys, version B, for a pair of enantiomeric reactions. a**. Short RInChIKey = bSA-BEANN-CPQZBLWAMR-DVCHMHGSMQ-EANNATPGMB-**MIILF-MCLVE**-NEANN. **b**. Short RInChIKey = bSA-BEANN-CPQZBLWAMR-DVCHMHGSMQ-EANNATPGMB-**MDSDX-MDUXS**-NEANN.

Version B again includes directionality in block 2 indicated by the first character (see section Version B) and reflects on the layered character of the RInChI by separating the InChIs into major and minor parts. The major parts shown in blocks 3, 4 and 5 represent separately hashed layers for chemical formula, connectivity, hydrogen and charge for the three groups of molecules in the RInChI. Note that block 5 is the same for both, as it is empty for both. The three following blocks are derived from the structures of minor layers with the first character of each block indicating the level of protonation. The two enantiomers in Figure 4 now differ only in the blocks 6 and 7 (highlighted) which include information about the stereochemistry.

Since RInChIKeys omit a large amount of information, it must be possible for different reactions to have the same RInChI keys. However, the chances of this are very low. Only two InChIKey clashes have been reported [9-12], despite the huge number of InChIKeys that have been generated. The RInChIKey is larger than the InChIKey and so the proportion of clashes should be correspondingly lower. Clashes, therefore, are likely to be exceedingly infrequent, but it is important to bear in mind that they are possible.

## Conversions

**Figure 5:**
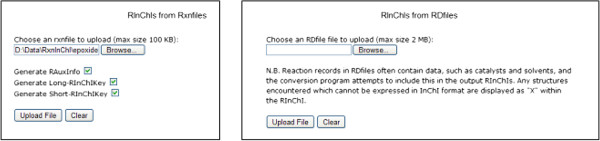
Web-based tools for the conversion of RxnFiles and RDFiles to RInChIs.

The algorithms for the conversions of Rxnfiles or RDfiles to RInChIs or RInChIKeys are Python scripts. The InChI-to-InChIKey algorithm, available within the official InChI software [1], was modified to a Python implementation to facilitate integration. Using the web-based conversion tools (Figure 5) on the RInChI website at http://www-rinchi.ch.cam.ac.uk, the conversion can easily be carried out.

### Generation of a RInChI from a Rxnfile and reverse conversion

**Figure 6:**
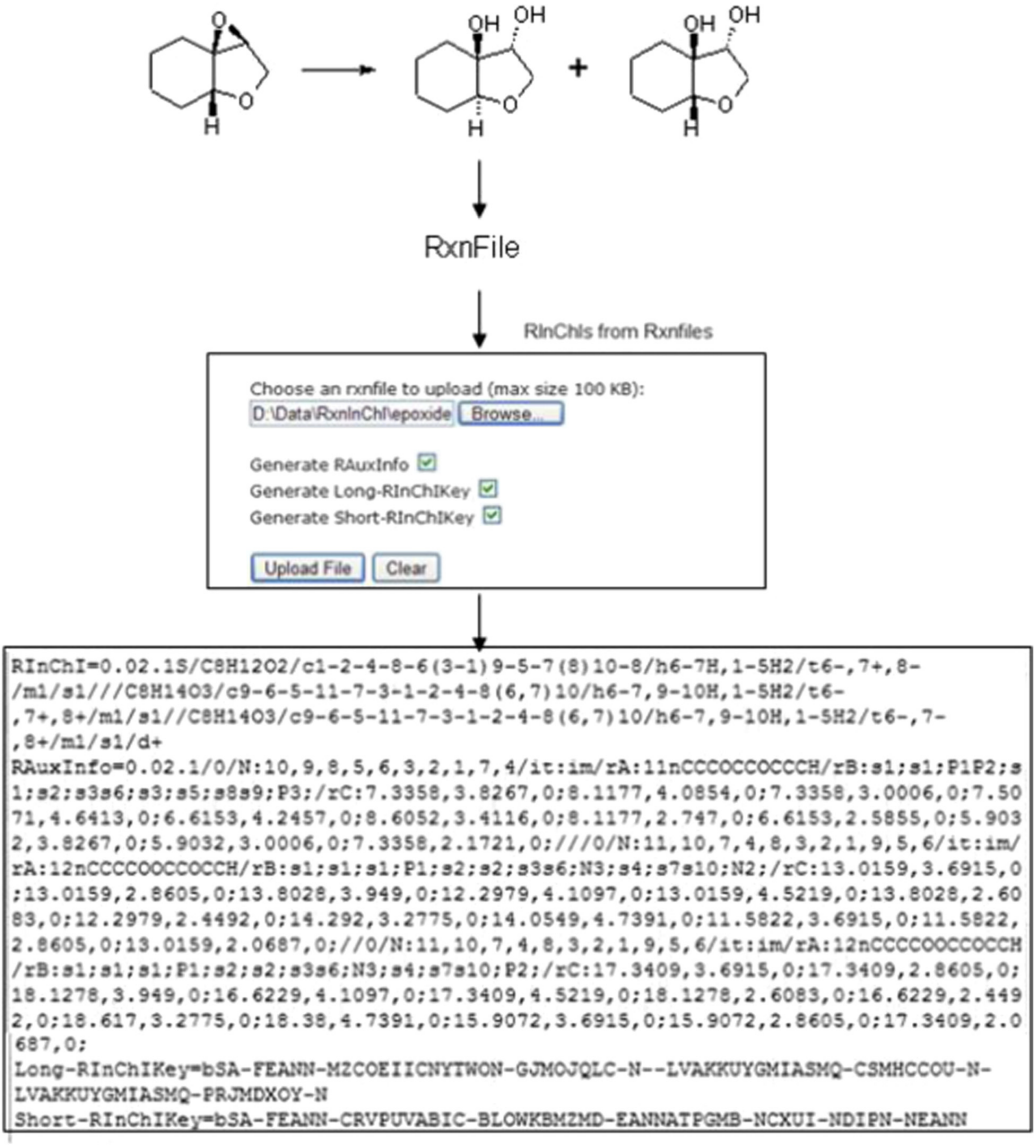
Conversion of a sample Rxnfile to a RInChI and RInChIKeys using the RInChI website tool.

A sample conversion is shown in Figure 6. After generating and saving a Rxnfile from a structural reaction diagram, the file is uploaded for conversion on the RInChI website. Users then have several options to choose from. They can generate the basic RInChI, add the long and short RInChIKey and fill in auxiliary information.

**Figure 7:**
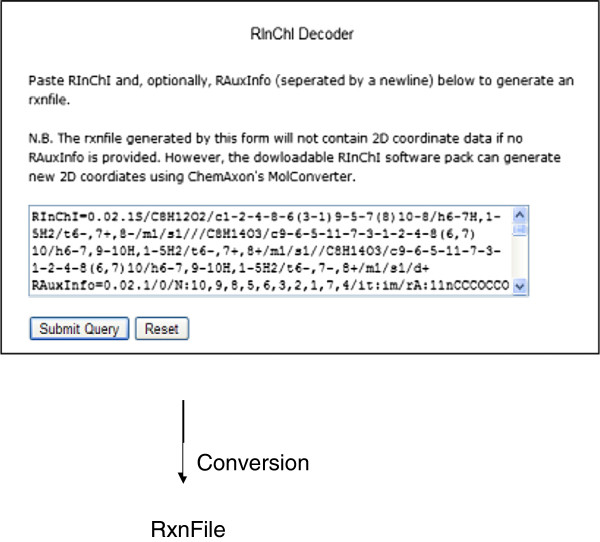
Web-based tool for decoding a RInChI to a Rxnfile.

In the reverse order, a RInChI can be converted to a Rxnfile and the corresponding reaction sketch using the Decoder (Figure 7) tool of the website. RAuxInfo data have to be provided if the Rxnfile should contain 2D coordinates. If this information is not available, ChemAxon’s MolConverter, provided with the RInChI software package must be used.

*A referee has pointed out that the order of sorting the reactants/products can depend on the minor layers of the InChI, and so a small change in the minor layers of a molecule can have a dramatic effect on the InChI key. This could be addressed by sorting first on major layers and then on minor layers. We intend to address this issue in a future version of the RInChI protocol.

### Generation of RInChI from RDfile

**Figure 8:**
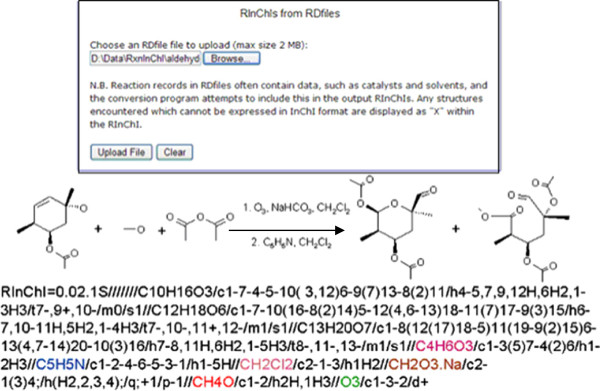
Conversion of a sample RDfile into a RInChI.

The conversion of large reaction databases to the corresponding database of RInChIs is fast and reduces the size of the database by about 90% by eliminating most non-relevant information. The conversion script extracts from a large RDfile the embedded rxnfiles and the molfiles representing agents, catalysts and solvents. The latter information is of special interest for identifying variations of a given core reaction. Therefore, the program generates as many Rxnfiles from a reaction as there are variations. An example for the conversion is shown in Figure 8, again using a web-based conversion tool

RInChI databases can be easily manipulated (see Section RInChI Applications) for analysis. For example, databases from different sources can be checked for duplicate reactions, for reactions using the same starting material or yielding the same product.

## RInChI applications

### Generation of a RInChI for multistep reactions

**Figure 9:**
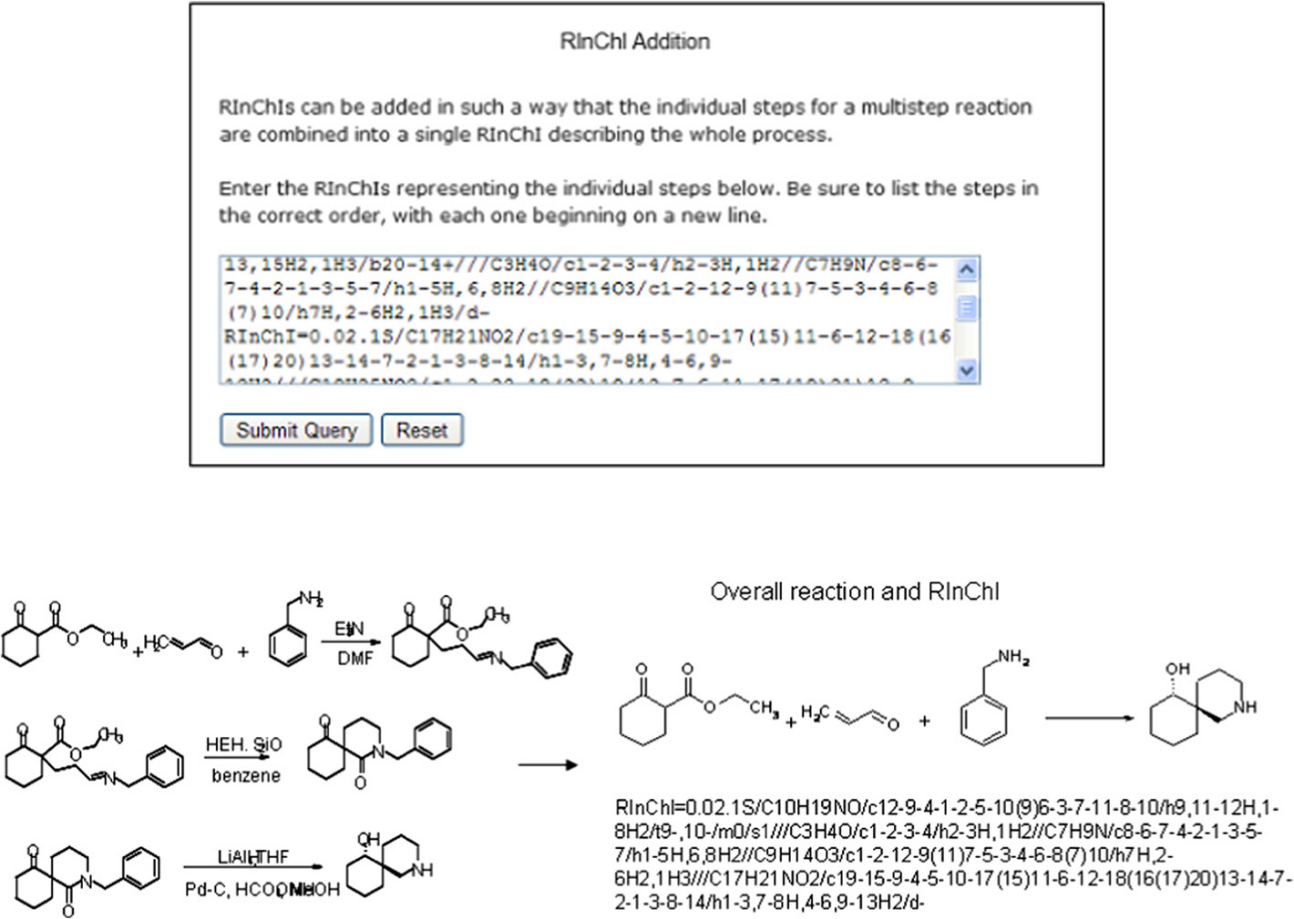
Generation of a RInChI for an overall reaction (multistep reaction).

The web-based tool (Figure 9) allows the formation of a summary RInChI for multistep reactions from the RInChIs of the individual steps. The RInChIs of each of the reactions have to be generated separately and added into the box in the correct sequential order. The Python script then produces a RInChI for the overall reaction that shows the initial starting material(s), the final product(s) and any starting material(s) or product(s) in the sequence of reactions that have not been changed. Some detailed information about each step is lost when multiple steps are combined, and the resultant RInChI cannot distinguish between reagents, solvents and catalysts in intermediate steps of the overall process.

### RInChI tools for analysis

Because of their smaller size as compared to RDfiles while still containing all essential chemical information, RInChI databases are very well suited for large-scale analysis. At the writing of this note, substance searching and changes in stereochemistry and rings have been implemented as Python scripts to exemplify the potential of RInChIs. The analyses can easily be carried out using the program’s website.

#### Searching for reaction partners

**Figure 10:**
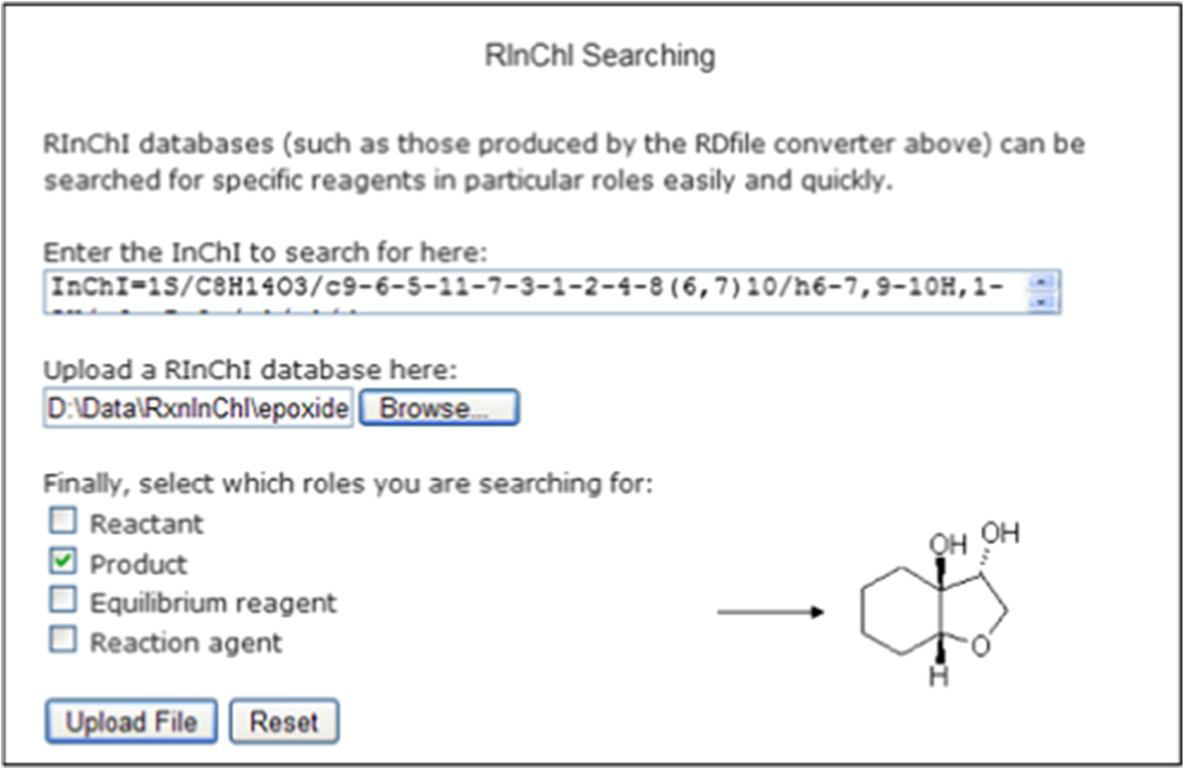
Web-based searching for a product (shown) in RInChI database.

RInChI databases can be searched for compounds taking part in a reaction as reactant, product, agent or equilibrium agent. For searching the database for the benzofuran derivative shown in Figure 10 as a product, the InChI notation of the compound and the RInChI database to be searched have to be entered into the respective boxes on the website. The result is a list of RInChIs of reactions that produce the benzofuran derivative. From this list the individual Rxnfiles and, subsequently, the structural diagram of the reactions can be generated *via* the RInChI decoder utility (Figure 7).

#### Structural analyses

The potential of analyzing RInChIs is further demonstrated by two preliminary analytical web-based tools which have been implemented in the RInChI program for certain structural changes in molecules participating in a reaction. However, their full application is limited by the lack of stoichiometric information in RInChIs.

**Figure 11:**
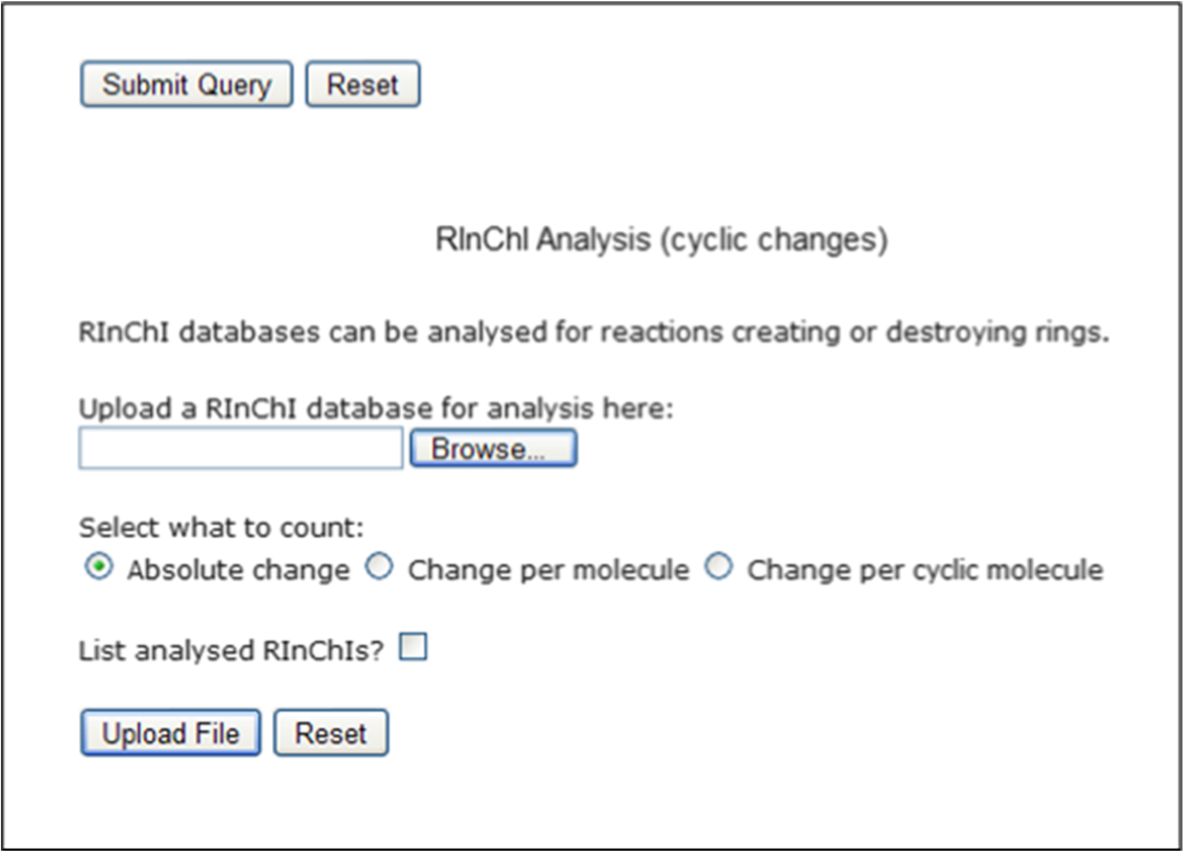
Analysis of cyclic molecules in a reaction.

One script searches a RInChI database for reactions in which the number of rings on either side of the reaction changes. Additionally, it is possible to count the change in rings per molecule or rings per cyclic molecule. This tool is based on the information entailed in the connectivity layer of the individual InChIs within a RInChI (Figure 11).

**Figure 12:**
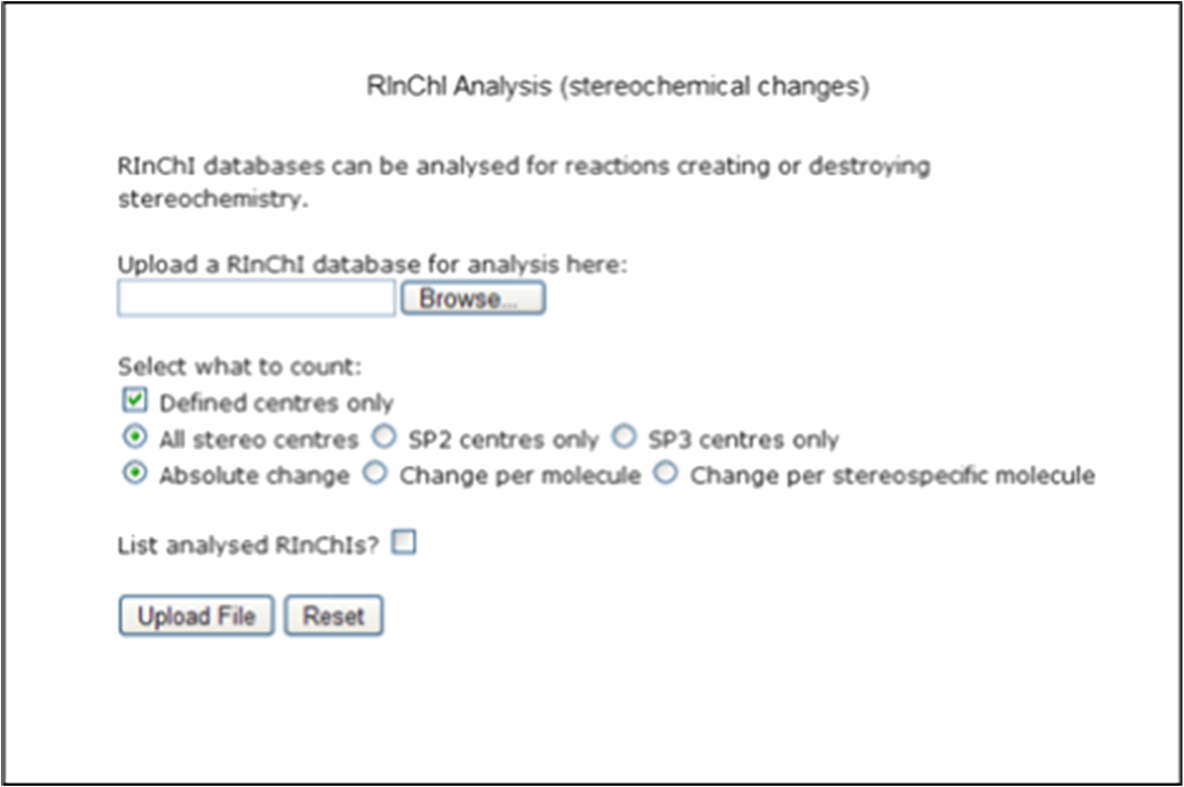
Stereochemical analysis of RInChIs.

The second tool analyses the stereochemical information in the layers of individual InChIs within a RInChI to calculate the changes in the number of stereocenters per molecule in a reaction (Figure 12).

### Database analysis

In order to further these goals, four large RDfiles containing nearly three thousand reactions, provided by Elsevier [13], FIZ Chemie Berlin [14], and InfoChem [15], were used for testing. With the large database of RInChIs generated from these files, much more information on the strengths and weaknesses of the format could be gleaned and general tools for RInChI manipulation developed.

These data sets were processed to generate 2900 RInChIs. The process took a few minutes on a desktop computer. Most of the computer time was required for generating InChIs from the structures in the RDfiles.

The file size was reduced by a factor of thirty moving from RDfiles to RInChI. Although 97% of the size was lost, most reaction data were retained. By removing a lot of information without chemical relevance, such as Cartesian coordinates, it is possible to manipulate and search the rest very quickly, using simple unix commands.

This database of RInChIs could be analyzed very rapidly using simple text-handling tools. Sorting the list showed that there were 298 duplicates. These turned out to be very similar processes which were distinguished only by free-text comments in the RDfiles. They were slightly different, therefore, but not different enough to have distinct RInChIs. The RInChI file contained 2602 unique reactions, in which 7342 molecules were present. Comparing these molecules across the whole file showed that 5240 of them were unique. It was possible to quickly identify the examples for which the same starting materials led to different products and different starting materials led to the same products. Although this fairly small database did not lead to any startling new discoveries, it illustrates how large amounts of chemical data can be compressed and analyzed effectively and cheaply with scalability to much larger systems.

## Conclusion

This note outlines the initial development of a program to generate the non-proprietary International Identifier for Reactions (RInChI). The identifier describes chemical reactions in a unique, freely-available and machine-readable character string that can be used both in printed and electronic data sources. The program is an extension of the IUPAC InChI project. A software package has been developed to generate RInChIs and RInChIKeys from Rxnfiles and RDfiles and to regenerate Rxnfiles from RInChIs. The package also includes several scripts to analyze databases for certain reaction participants and structural changes in rings or in stereochemistry. All tools are web-based and are available on the project’s website at http://www-rinchi.ch.cam.ac.uk. The individual web-based tools on the website are shown in the figures together with relevant examples. Further work on the project under the supervision of the InChI Trust is continuing.

## Competing interest

The authors declare that they have no competing interest.

## Authors’ contributions

The programming work was carried out by CA under the supervision of JG at Cambridge University. GG drafted the manuscript and led the RInChI Working Group under the supervision of the IUPAC Division VIII Subcommittee and the InChI Trust. All authors read and approved the final manuscript.
